# The Sacred Ibis debate: The first test of evolution

**DOI:** 10.1371/journal.pbio.2005558

**Published:** 2018-09-27

**Authors:** Caitlin Curtis, Craig D. Millar, David M. Lambert

**Affiliations:** 1 Environmental Futures Research Institute, Griffith University, Nathan, Queensland, Australia; 2 Centre for Policy Futures and School of Biological Sciences, University of Queensland, Brisbane, Queensland, Australia; 3 School of Biological Sciences, University of Auckland, Private Bag, Auckland, New Zealand

## Abstract

In 1798, Napoleon Bonaparte’s army invaded Egypt, returning with many treasures including large numbers of Sacred Ibis mummies. The ancient Egyptians revered the ibis and mummified literally millions of them. The French naturalist Georges Cuvier used these mummies to challenge an emerging idea of the time, namely Jean-Baptiste Lamarck’s theory of evolution. Cuvier detected no measurable differences between mummified Sacred Ibis and contemporary specimens of the same species. Consequently, he argued that this was evidence for the “fixity of species.” The “Sacred Ibis debate” predates the so-called “Great Debate” between Cuvier and Geoffroy Saint-Hilaire and the publication of Darwin’s *On the Origin of Species* five decades later. Cuvier’s views and his study had a profound influence on the scientific and public perception of evolution, setting back the acceptance of evolutionary theory in Europe for decades.

## Introduction

When Napoleon’s army invaded Egypt in 1798, his soldiers collected many treasures. The most famous of these was the Rosetta Stone discovered by a French soldier in 1799. This tablet comprised three versions of a decree that eventually enabled the deciphering of Egyptian hieroglyphics [1]. Ironically, the stone was captured by the British at the French surrender of Alexandria and never made it to French soil. It has been in the British Museum since 1802. Of almost equal fascination at the time was the large number of animal mummies that were brought back to France as “spoils of war.” Many species, including cats, jackals, dogs, crocodiles, snakes, ibis, and other birds, as well as human mummies, were described in exquisite detail in the *Description de l’Égypte* (1809–1829). Like the Rosetta Stone, many of these mummies were deposited in museums.

**Fig 1:**
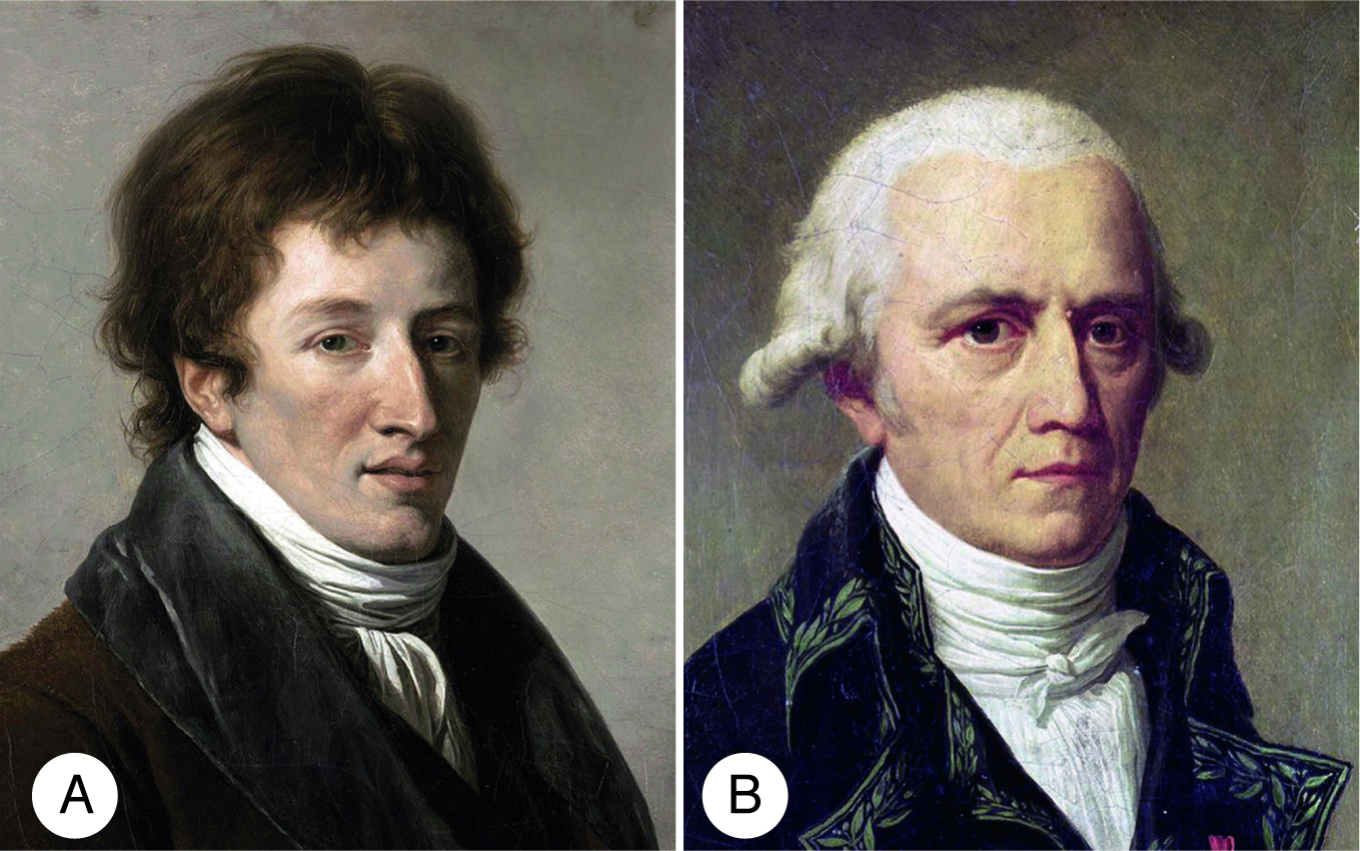
The two central figures in the first test of evolution. (A) Georges Cuvier (1769–1832) and (B) Jean-Baptiste Lamarck (1744–1829).

These mummies captivated the imagination of the public. Numerous “unwrappings” of human and animal mummies took place, including several ibis [2, 3]. The Egyptians mummified literally millions of these birds and stored them in vast underground catacombs [2]. In addition to the extensive military forces, the French invasion included a remarkable delegation of more than 150 civilian intellectuals, called savants, including Geoffroy Saint-Hilaire, who would later become an important figure in the development of evolutionary thought. The savants established the Egyptian Institute (*Institut d’Égypte*) and went on to painstakingly collect and document the physical, natural, and cultural history of the region. The Sacred Ibis (*Threskiornis aethiopicus*) mummies ignited a fierce debate about the reality of evolution between two of the giants of 19th century natural history, Georges Cuvier and Jean-Baptiste Lamarck (Fig 1). This debate preceded the “Great Debate” and occurred decades before the publication of the pivotal works of Charles Darwin and Alfred Russel Wallace on natural selection and evolution.

## Mistaken identity: Ibis mummies were misidentified as “storks”

**Fig 2:**
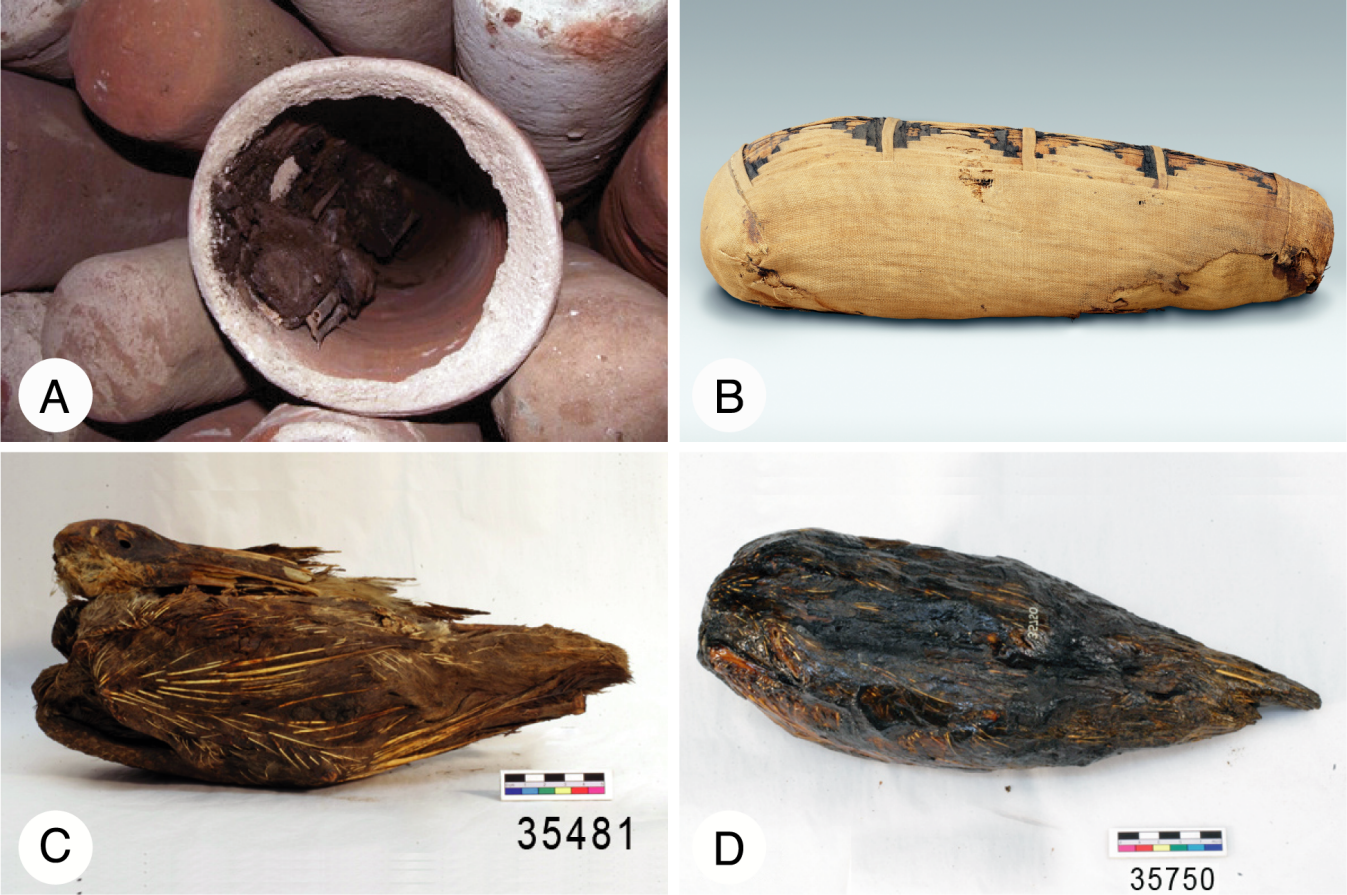
Mummified Sacred Ibis. (A) Empty and full pottery vessels from catacombs from Saqqara, Egypt (*photo credit Sally Wasef*), (B) mummified Sacred Ibis wrapped in cloth (*photo credit Metropolitan Museum of Art*, *New York*), (C) a well-preserved example of an unwrapped Sacred Ibis mummy (the head and wings of the bird are clearly visible), and (D) a mummified Sacred Ibis dipped in resin.

In Egypt, animal mummies vastly outnumbered those of humans [4]. The catacombs at Tuna el-Gebel in Egypt, for example, are estimated to contain 4 million Sacred Ibis mummies [5], many of which are well preserved (Fig 2). Ancient Egyptians revered the Sacred Ibis as a manifestation of Thoth, the god of wisdom and writing. Images of ibis were used in hieroglyphic writings and as amulets and statues representing Thoth. From the Late Period onward (ca. 7th century BC), these birds were mummified as offerings to Thoth. Generally, once killed, they were desiccated with salts and covered with oils and resins. The wrapped birds were then typically sealed in large pottery vessels—sometimes two or more to a pot (Fig 2). Others were placed in wooden coffins or covered with a layer of cartonnage (similar to “papier-mâché”) that was plastered and painted [4].

**Fig 3:**
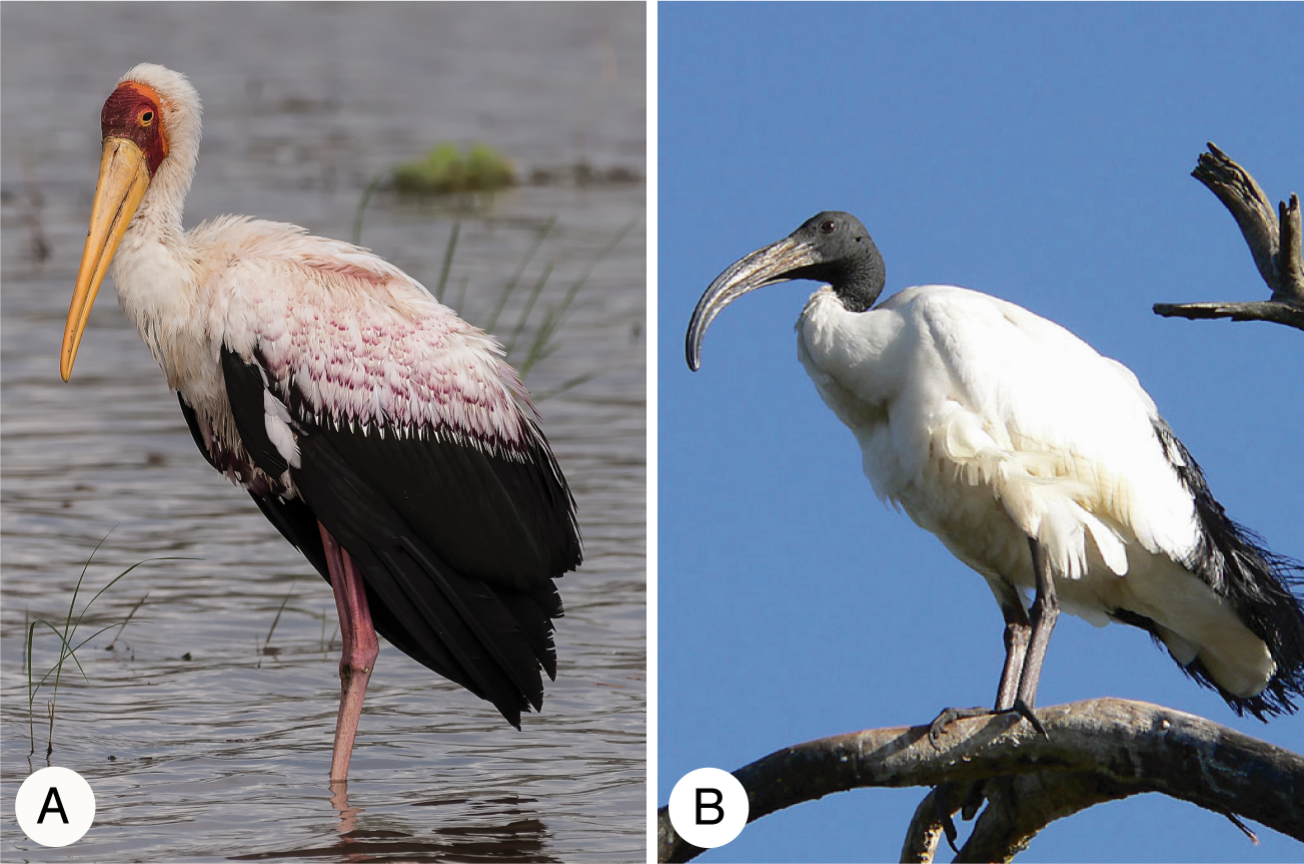
Stork and ibis. (A) Yellow-billed stork (photo credit Becky Matsubara) and (B) Sacred Ibis (photo credit Christiaan Kooyman).

By the late 18th century, most scholars mistakenly believed that the Sacred Ibis mummies were actually yellow-billed storks (then *Tantalus ibis*, now *Mycteria ibis)* [6] (Fig 3). This error is perhaps understandable given that Sacred Ibis populations were not present in Europe at the time. Therefore, 18th century specimens of Sacred Ibis in Europe were limited to a few unidentified birds in museums.

## Opposing views within the French National Museum of Natural History: Cuvier and Lamarck

While Napoleon’s Egyptian conquest included many prominent scientists, the French naturalist Georges Cuvier chose to remain in Paris at the French National Museum of Natural History, where he would later very publicly argue his opposition to evolution. At the time of the Egyptian conquest, however, Cuvier was developing his principle of the “correlation of parts.” Primarily, Cuvier believed that an organism’s parts were perfectly adapted and linked in such a way that any modification to one of the parts would prevent the survival of the organism as a whole. The correlation of parts was so pervasive and powerful that Cuvier believed it could be used to predict the function of any particular part as well as its relationship to the organism as a whole. Using this principle, Cuvier proclaimed to be able to predict the entire form of any organism from mere fragments of bones or a few organs—including reconstructing entire skeletons of extinct creatures from fossilized bones.

Furthermore, Cuvier’s belief in the correlation of parts led him to argue for the “fixity of species” or the idea that each species is based on an ideal form that cannot change over time [7]. Though Cuvier was unyielding in his belief that species were unchanging and did not evolve, he rightly argued that extinctions had been widespread throughout the Earth’s geological history. Cuvier recognized the existence of fossilized species for which no modern relative existed, a revolutionary idea at the time. However, Cuvier rejected the idea that such fossilized remains could have been the ancestors of living forms [8].

Cuvier’s idea of the “fixity of species” was in conflict with the views of Lamarck, his contemporary at the museum. However, Lamarck’s prestige and political influence was modest in comparison to Cuvier’s ever-increasing prominence, intellect, and showmanship. These attributes contributed to his notoriety in the scientific and popular culture of the time. In contrast to the “fixity of species,” Lamarck argued for a continuous slow transmutation of animal species over time (now known as “phyletic gradualism”) [9]. In Lamarck’s theory of transmutation (or species mutability), species would proceed up the “Great Chain of Being” [10] from simple to complex, with humans at the pinnacle. Lamarck’s *Philosophie Zoologique* [9]—published half a century before Darwin’s *On the Origin of Species*—included the following ideas: species change through evolutionary time; evolutionary change is slow and imperceptible; evolution occurs through adaptation to the environment; it generally progresses from the simple to the complex, although in a few cases, it proceeds in reverse; and species are related to one another by common descent. Furthermore, Lamarck’s theory incorporated the fact that the world is old and proposed that life was a result of abiogenesis, i.e., the origin of life derives from inanimate matter [11].

## Cuvier performed the first test of evolution

Cuvier had the opportunity to study two Sacred Ibis mummies that were collected by Saint-Hilaire, who also worked at the National Museum of Natural History in Paris. Their coloration matched that of the yellow-billed stork: white plumage and wing feathers marked with black, but the bones were too small to be a stork and the shape of the beak was wrong—it was curved, not straight like that of a stork (Fig 3). One conceivable explanation for these differences was evolution: namely, that “storks” had morphologically changed since the time of the Egyptians [6].

As more mummies were brought back from Egypt, Cuvier’s assistant Rousseau was able to assemble a composite skeleton. This skeleton remains on display at the museum. Cuvier used this skeleton (and other loose bones from mummies) to make many careful morphometric measurements of the mummified birds. According to Cuvier, there had been no changes in the morphology of the Egyptian mummified “stork” over time. Cuvier compared the mummified bones to skeletons of six avian specimens of another species with similar general characteristics. These specimens had an equivalent coloration, body size, and, most importantly, a curved beak. He included two known stork specimens (*M*. *ibis)* in the analysis. Cuvier carefully recorded body part measurements from all of these birds.

Based on these measurements, Cuvier correctly established that the mummies were not storks. He determined that the mummified birds matched the unclassified birds from the museum. Cuvier went on to name these birds *Numenius ibis*, and they have subsequently been reclassified as *T*. *aethiopicus* (Sacred Ibis). Cuvier also recovered a few uniquely shaped black feathers from a mummy that provided further evidence for his identification of the mummified birds as ibis. To Cuvier’s knowledge, these distinctive black feathers were a characteristic of the genus *Numenius*. Cuvier preserved these feathers for future examination as “a remarkable monument of antiquity and a peremptory proof of the identity of species” [12].

The measurements of the mummified bones were not a perfect match with those taken from the museum specimens of Sacred Ibis. However, the measurements between the ancient material and the then-contemporary Sacred Ibis were similar, and Cuvier concluded that no detectable anatomical changes had occurred over time. This made him the first to test the idea of evolution.

## Sacred Ibis mummies become the focus of evolutionary debate

Lamarck and Cuvier publicly presented the animal mummies to the French Academy in 1802, along with Comte de Lacépède [13]. Referring to the mummies, the latter author remarked, “these animals are perfectly similar to those of today” [14]. Cuvier also described the lack of change in the ibis mummies as follows: “We certainly do not observe more differences between these creatures and those which we see today than between human mummies and today’s human skeletons.” [12]. Whilst Cuvier and Lamarck agreed in their presentation to the Academy that no discernible changes had taken place since the time of the Egyptians, their opposing views regarding the “fixity of species” led them to clash on the significance of the findings. Lamarck insisted that extensive periods of time with changing environmental conditions would be required to see the slow, gradual changes (i.e., transmutations) in organisms over time [9]. Lamarck’s argument was that a passage of 3,000 years would have been insufficient to observe evolutionary processes because the environmental conditions in Egypt had not changed during this time. According to Lamarck, “It would indeed be very odd if it were otherwise; for the position and climate of Egypt are still very nearly what they were in those times. Now the birds which live there, being still in the same conditions as they were formerly, could not possibly have been forced into a change of habits” [9]. Cuvier acknowledged that only 2,000 to 3,000 years had elapsed at most (this estimate was recently shown to be accurate [15]), but he denied that evolution would result from longer periods of time. He argued that longer timescales simply contain the sum of changes within shorter periods. In other words, he reasoned that since no changes had been observed over approximately 3,000 years, it was unreasonable to argue that any longer timescale would produce them. Cuvier went on to publicly argue that his study on the Sacred Ibis was evidence for the “fixity of species” in opposition to Lamarck. Throughout his career, he produced several increasingly refined iterations of his case study of the mummified ibis that were published from the late 18th century to at least 1827. Cuvier even carried his argument through to Lamarck’s death, incorporating it into his spiteful eulogy for Lamarck [16].

## The Great Debate

**Fig 4:**
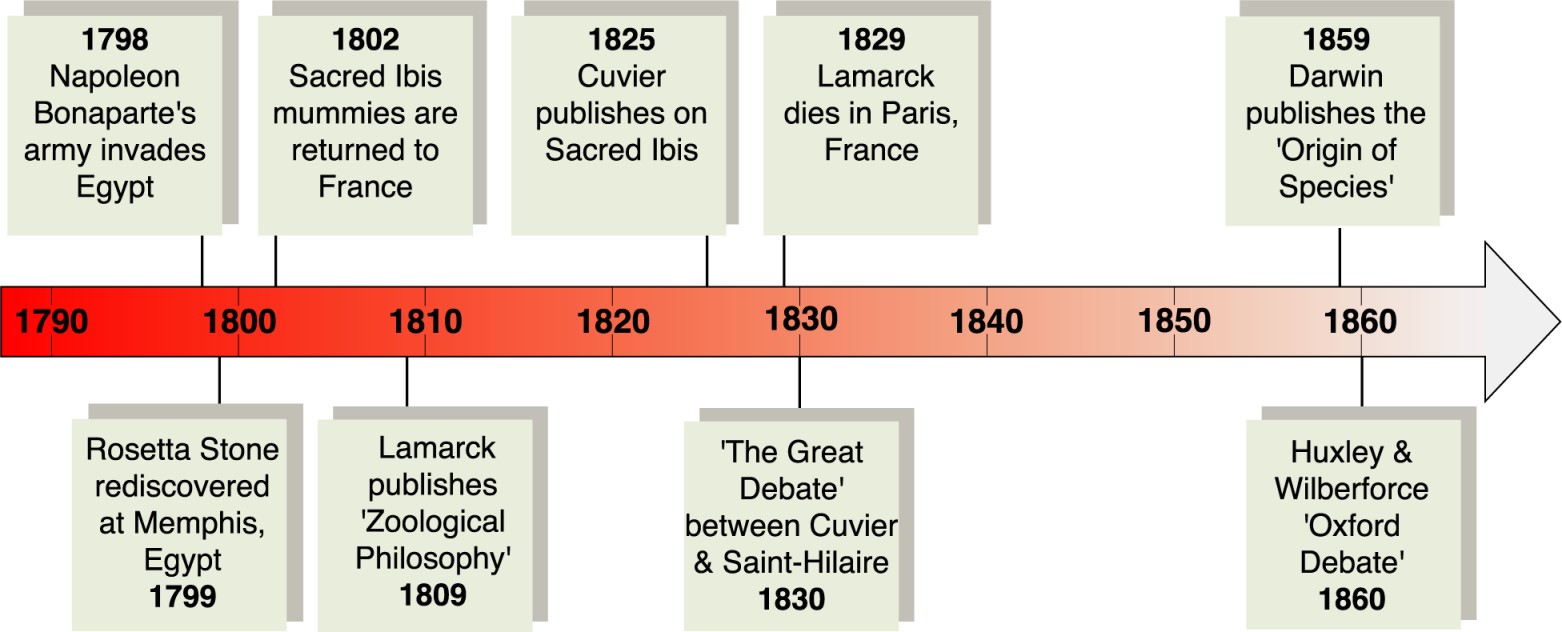
A timeline showing some of the major events in the history of the first test of evolution.

The Sacred Ibis debate set the scene for a much more intense controversy. A year after Lamarck’s death in 1829, a heated debate ensued in the French Academy of Sciences. This is commonly referred to as “The Great Debate” (Fig 4) (as distinct from the later debate between Huxley and Wilberforce). The debate was a broad philosophical exchange about the importance of functional properties of organisms and the “fixity of species”, what we now know as evolution. As in the Sacred Ibis debate, Cuvier was one of the central participants, but on this occasion, Cuvier’s protagonist was Saint-Hilaire. Saint-Hilaire generally supported Lamarck’s evolutionary ideas but emphasized organisms as products of the laws and principles of biology. Cuvier argued that the functional properties of organisms explained their existence through the will of a divine creator.

## Conclusion

The case of the Sacred Ibis highlights the disproportionate influence that a charismatic and dominant personality like Cuvier can have. The magnitude of Cuvier’s influence has been the subject of discussion [17], but, as Burkhardt [18, 19] argued, “…Cuvier’s magisterial and disapproving presence has long been recognised as a factor in the poor reception of Lamarck’s evolutionary theory by his contemporaries.” Cuvier’s unwillingness to consider the potential for very small differences to accumulate over much longer timeframes enabled him to interpret his study in a way that supported his own beliefs and set back the acceptance of evolution for the next five decades [6]. The debate about the Sacred Ibis is an important but often unrecognized episode in the history of science. Of great importance is the reminder, even today, of the power of a strong personality and that the belief in “what they know to be true” can dramatically influence the direction of science and public opinion.

## References

[1] Ray J. The Rosetta Stone and the rebirth of Ancient Egypt. London: Profile Books; 1996.

[2] Taylor JH. The collection of Egyptian mummies in the British Museum: Overview and potential for study In: Fletcher A, Antoine D, Hill JD, editors. Regarding the Dead: Human Remains in the British Museum. London: British Museum Press; 2014 pp. 103–114.

[3] Pearson J. Some account of two mummies of the Egyptian Ibis, one of which was in remarkably perfect state. J Philos Trans R Soc Lond. 1805; 95**:** 264–271.

[4] Ikram S. Divine creatures: Animal mummies in ancient Egypt Cairo: American University in Cairo Press; 2005.

[5] El Mahdy C. Mummies, myth and magic in ancient Egypt London: Thames and Hudson;1989.

[6] Rudwick MJS. Bursting the limits of time Chicago: The University of Chicago Press; 2005.

[7] Coleman W. Georges Cuvier, Zoologist: A Study in the History of Evolution Theory. Cambridge, Massachusetts: Harvard University Press; 1964.

[8] Honeywill R. Lamarck’s evolution Two centuries of genius and jealousy. Sydney: Murdock Books; 2008.

[9] Lamarck J-B. Zoological Philosophy: An exposition with regard to the natural history of animals. United States of America: University of Chicage Press; 1984.

[10] Lovejoy AO. The great chain of being: A study of the history of an idea Boston: Harvard University Press; 1936.

[11] Graur D, Gouy M, Wool D. In retrospect: Lamarck’s treatise at 200. Nature. 2009; 460: 688–689. 10.1038/460688a

[12] Cuvier G. Discourse on the revolutionary upheavals on the surface of the globe and on the changes which they have produced in the animal kingdom [Translated by Ian Johnston] Arlington: Richer Resources; 2009.

[13] Lacépède B-G-E, Cuvier G, Lamarck J-B. Rapport des professeurs du Muséum sur les collections d'histoire naturelle rapportées d'Égypte. Annales du Museum d’Histoire Naturelle. 1802; 1: 234–241.

[14] Burkhardt RW. Lamarck, evolution, and the inheritance of acquired characters. Genetics. 2013; 194: 793–805. 10.1534/genetics.113.151852 23908372 PMC3730912

[15] Wasef S, Wood R, Ikram S, Curtis C, Holland B, Willerslev E, Millar C, Lambert DM. Radiocarbon dating of Sacred Ibis mummies from ancient Egypt. J Archaeol. Sci. 2015; 4: 355–361.

[16] Cuvier G. Elegy of Lamarck. New Philos J Edinb. 1836; XX: 1–22.

[17] Corsi P. The revolutions of evolution: Geoffroy and Lamarck, 1825-1840. Bulletin du Musée d’Anthropologie Préhistorique de Monaco. 2012; 51: 97–122.

[18] Burkhardt W. Lamarck, evolution, and the politics of science. J Hist Biol. 1970; 3: 275–298. 11609655 10.1007/BF00137355

[19] Burkhardt RW. The Leopard in the garden: life in close quarters at the Muséum d’Histoire Naturelle. Isis. 2007; 98: 675–694. 18314641 10.1086/529263

