# Correction: The Sacred Ibis debate: The first test of evolution

**DOI:** 10.1371/journal.pbio.3000052

**Published:** 2018-10-23

**Authors:** The PLOS Biology Staff

## Notice of republication

This article was republished on October 10, 2018, to correct a figure error that was introduced by PLOS staff during the typesetting process. Fig 3B contained an image that was not available for publication under the Creative Commons Attribution License (creativecommons.org/licenses/by/4.0/). Fig 3B has therefore been replaced with an image in the public domain. The caption remains the same.

The publisher wishes to apologize to the authors and readers for this error. Please download this article again to view the correct version.
